# Tobacco Use Among Tribal Communities in India: A Systematic Review and Meta-Analysis of Prevalence Studies

**DOI:** 10.7759/cureus.87874

**Published:** 2025-07-14

**Authors:** Santanu Nath, Venkata Lakshmi Narasimha, Bijit Biswas, Ujjwal Kumar, G Jahnavi, Arshad Ayub, Benazir Alam, Niwedita Jha, Vinayagamoorthy Venugopal, Sudip Bhattacharya, Rana Jugdeep Singh, Saurabh Varshney

**Affiliations:** 1 Psychiatry, All India Institute of Medical Sciences, Deoghar, IND; 2 Psychiatry, Centre for Addiction Medicine, National Institute of Mental Health and Neurosciences, Bengaluru, IND; 3 Community and Family Medicine, All India Institute of Medical Sciences, Deoghar, IND; 4 Vital Strategies Tobacco Control Project, All India Institute of Medical Sciences, Deoghar, IND; 5 Tobacco Control, Vital Strategies, New Delhi, IND; 6 Otolaryngology, All India Institute of Medical Sciences, Deoghar, IND

**Keywords:** india, prevalence, smokeless tobacco, smoking tobacco, systematic review and meta analysis, tobacco, tribal communities

## Abstract

Tribal populations in India face multiple health challenges, including high tobacco use. However, national-level estimates specific to tribal communities are lacking. This systematic review and meta-analysis assessed the prevalence of tobacco use among tribal populations in India, disaggregated by gender and type of tobacco product.

Following the Preferred Reporting Items for Systematic Reviews and Meta-Analyses (PRISMA) 2020 guidelines, four databases, PubMed, Embase, Scopus, and Web of Science, were searched up to 31 January 2024. Studies reporting tobacco use among tribal populations in India were included. Meta-analysis was performed using a random-effects model. Subgroup analyses were conducted by gender and tobacco type. Heterogeneity was assessed using the I-squared (I²) statistic. Publication bias was assessed using the deviation from ordinary least squares (OLS) regression line (DOI) plots and the Luis Furuya-Kanamori (LFK) index.

Thirty-nine studies involving 56,883 tribal individuals were included. The pooled prevalence of tobacco use was 60% (95% confidence interval (CI): 49%-70%; I² = 99.6%). Among male individuals, prevalence was 66% (95% CI: 42%-83%; I² = 99.2%), and among female individuals, it was 42% (95% CI: 22%-66%; I² = 99.6%). The prevalence of smokeless tobacco use was 45% (95% CI: 30%-62%) versus 20% for smoked tobacco (95% CI: 14%-27%). Nine studies lacked separate data on smoked and smokeless forms. The LFK index score of 0 indicated no asymmetry in the DOI plot, suggesting an absence of publication bias among the included studies. The majority of studies demonstrated a low risk of selection bias (78.5%), whereas fewer studies showed a low risk of information bias (57.1%) and reporting bias (53.5%).

Tobacco use among tribal populations in India is alarmingly high, especially among men and for smokeless products. These findings warrant urgent, culturally appropriate public health interventions.

## Introduction and background

India is home to over 104 million tribal individuals who represent one of the most socio-culturally distinct and vulnerable population groups [1]. These communities face a quadruple burden of diseases comprising communicable diseases, non-communicable diseases, malnutrition, mental illness, and addiction. Their unique socio-economic and cultural contexts, along with limited access to health services, place them at particular risk-especially amidst rapid urbanization. Mental health and substance use, particularly tobacco addiction, are recognized as key public health challenges and important barriers to achieving the 2030 Sustainable Development Goals (SDGs) [1,2].

Tobacco is the most widely used psychoactive substance in India. The National Mental Health Survey (2015-16) reported that 22.4% of individuals used psychoactive substances, with tobacco accounting for 20.9% and a treatment gap of 91.8% [3]. The Global Adult Tobacco Survey - Phase 2 (2016-17) reported an overall tobacco use prevalence of 28.6%, with 10.7% using smoked tobacco (ST) and 21.4% using smokeless tobacco (SLT) [4]. However, neither survey provides disaggregated data on tribal populations, leaving a major gap in understanding the national burden within these communities.

Multiple regional studies report high tobacco use among tribal groups, with SLT consistently more prevalent than smoked forms. Prevalence has ranged from 23% to over 90% across different states, with higher rates often observed among male users. Associations have also been documented between tobacco use and health outcomes such as pulmonary tuberculosis, poor oral hygiene, hypertension, and hazardous alcohol use [5-11].

In the absence of national pooled estimates, this systematic review and meta-analysis aim to quantify the prevalence of tobacco use among tribal populations in India by gender and tobacco type. Findings are expected to inform targeted public health strategies and contribute toward national tobacco control goals and the SDGs.

## Review

Search strategy

This systematic review and meta-analysis were conducted following the Preferred Reporting Items for Systematic Reviews and Meta-Analyses (PRISMA) 2020 guidelines [12]. The protocol was registered in the International Prospective Register of Systematic Reviews (PROSPERO; ID: CRD42024509184). A systematic search was performed across four electronic databases, PubMed, Embase, Scopus, and Web of Science, for studies published up to 31 January 2024. The search strategy included the Medical Subject Headings (MeSH) terms: “Tobacco” AND “Tribal” AND “India,” adapted for each database. Additionally, reference lists of eligible articles were screened for further relevant studies. Two reviewers independently screened titles and abstracts for relevance. Disagreements were resolved through discussion, and unresolved cases were adjudicated by a third reviewer.

Study's inclusion and exclusion criteria

**Figure 1:**
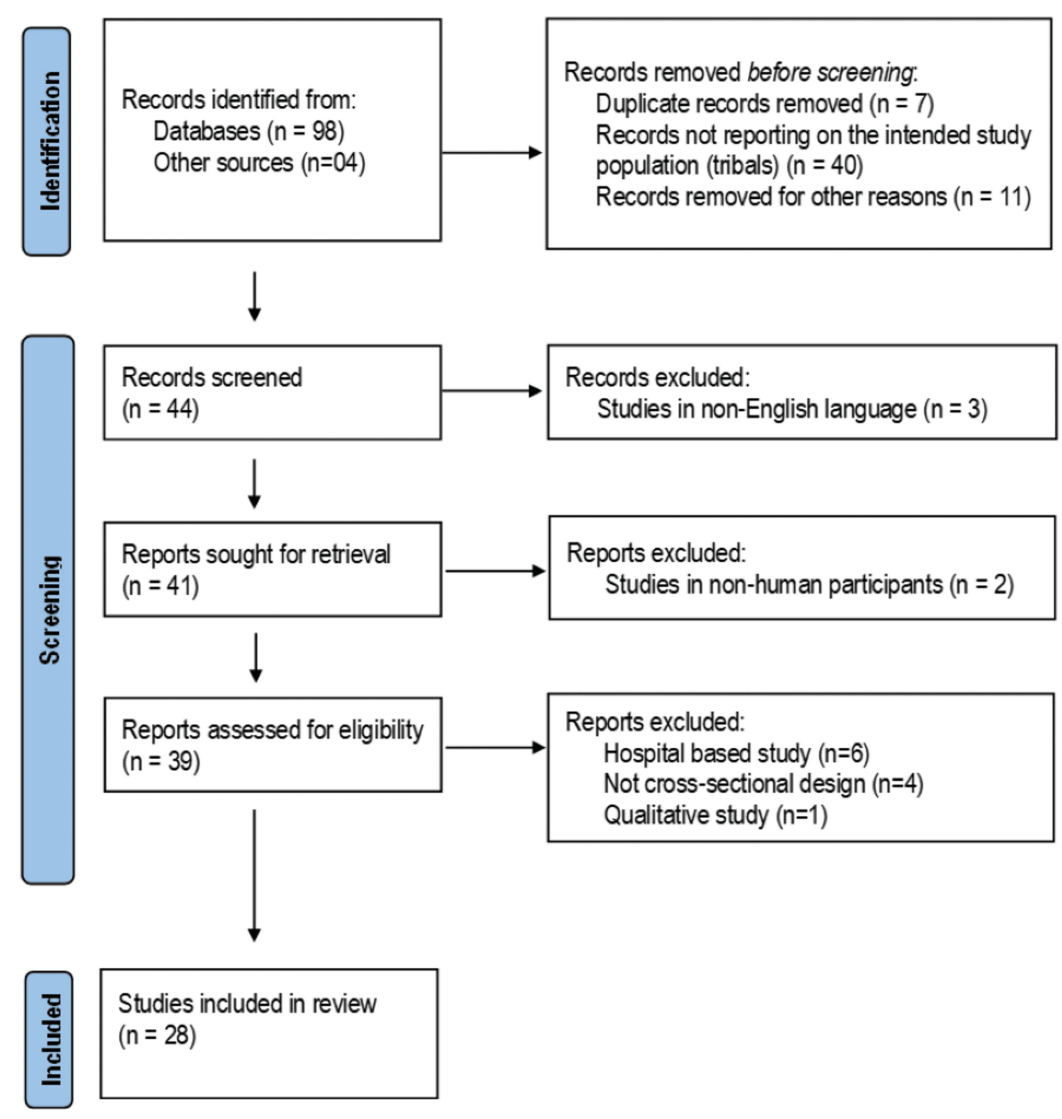
PRISMA 2020 Flow Diagram Showing Identification, Screening, and Inclusion of Studies PRISMA: Preferred Reporting Items for Systematic Reviews and Meta-Analyses

Studies were included if they reported the prevalence of any form of tobacco use (smoked, smokeless, or both) among tribal populations in India, were published in peer-reviewed journals, and were available in English. Only cross-sectional community-based surveys were considered. Studies involving non-human participants or not published in English were excluded. Full-text articles meeting inclusion criteria were retrieved and assessed in detail (Figure 1).

Data extraction and quality assessment

Four reviewers independently extracted data using a standardized Microsoft Excel (Microsoft Corporation, Redmond, USA) template. Extracted variables included author, year of publication, study location, study design, sample size and characteristics, tribal group, and prevalence of tobacco use. Data were also collected separately for smoked and smokeless tobacco and gender-specific prevalence where available.

Data analysis

Quality assessment of the included studies was independently conducted by two experts using the Joanna Briggs Institute (JBI) Critical Appraisal Tool for Prevalence Studies [13,14]. In cases of disagreement, a third senior expert reviewed the findings and provided the final decision. Risk of bias (ROB) and quality grading were further evaluated using Review Manager (RevMan) version 5.4 (Cochrane Interactive Learning, England and Wales), based on expert judgment [15]. Any discrepancies were resolved through consensus with a third senior reviewer. Meta-analysis was performed using R software (version 4.5.1; R statistical software (R Foundation for Statistical Computing, Vienna, Austria), employing the ‘meta’ and ‘glmm’ packages [16]. Pooled prevalence estimates were calculated using a random-effects model based on generalized linear mixed models (GLMM), implemented via Monte-Carlo likelihood approximation. Subgroup analyses were carried out by gender (male, female) and type of tobacco use (smoked, smokeless). Heterogeneity across studies was quantified using the I² statistic, with higher values indicating substantial between-study variation. Publication bias was assessed using the deviation from the ordinary least squares (OLS) regression line (DOI) plots and measured by the Luis Furuya-Kanamori (LFK) index [17].

**Table 1:** Quality Assessment of Included Studies Using the Joanna Briggs Institute (JBI) Critical Appraisal Tool for Prevalence Studies

Sl. No.	Authors and Year	Was the Sample Frame Appropriate to Address the Target Population?	Were Study Participants Sampled in an Appropriate Way?	Was the Sample Size Adequate?	Were the Study Subjects and the Setting Described in Detail?	Was the Data Analysis Conducted with Sufficient Coverage of the Identified Sample?	Were Valid Methods Used for the Identification of the Condition?	Was the Condition Measured in a Standard, Reliable Way for All Participants?	Was There Appropriate Statistical Analysis?	Was the Response Rate Adequate, and If Not, Was the Low Response Rate Managed Appropriately?
1	Agrawal et al. 2023 [5]	Yes	Yes	Yes	Yes	Yes	Yes	Yes	Yes	Yes
2	Babu et al. 2024 [18]	Yes	Yes	Yes	Yes	Yes	Yes	Yes	Yes	Yes
3	Bhar et al. 2019 [19]	Yes	Yes	No	Yes	Yes	Yes	Yes	Yes	Yes
4	Chellappa et al. 2021 [20]	No	No	No	Yes	Yes	Yes	Yes	Yes	Unclear
5	Doke et al. 2021 [21]	No	No	Yes	Yes	Yes	Unclear	Unclear	Yes	Yes
6	Doke et al. 2022 [22]	No	No	Yes	Yes	Yes	Unclear	Unclear	Yes	Yes
7	Ganie et al. 2020 [23]	Yes	Yes	Yes	Yes	Yes	Yes	Yes	Yes	Yes
8	Giri et al. 2022 [10]	Yes	Yes	Yes	Yes	Yes	Unclear	Unclear	Yes	Unclear
9	Gopalankutty et al. 2020 [24]	Yes	Yes	Yes	Yes	Yes	Unclear	Unclear	Yes	Unclear
10	Haque et al. 2021 [9]	Yes	Yes	No	Yes	Yes	Unclear	Unclear	Yes	Unclear
11	Kumar et al. 2019 [25]	Yes	Yes	Yes	Yes	Yes	Unclear	Unclear	Yes	Unclear
12	Kumar et al. 2022 [26]	Yes	Yes	Yes	No	Yes	Unclear	Unclear	Yes	Unclear
13	Kumar et al. 2022 B [27]	Yes	Yes	Yes	No	Yes	Yes	Yes	Yes	Unclear
14	Kumar et al. 2022 C [28]	No	No	Yes	No	Yes	Unclear	Unclear	Yes	Unclear
15	Kumaraguru et al. 2023 [29]	Yes	Yes	Yes	No	Yes	Unclear	Unclear	Yes	Unclear
16	Madhu et al. 2019 [7]	Yes	Yes	No	Yes	Yes	Yes	Yes	Yes	Yes
17	Meshram et al. 2023 [30]	Yes	Yes	Yes	Yes	Yes	Yes	Yes	Yes	Yes
18	Murmu et al. 2023 [31]	Yes	Yes	Yes	Yes	Yes	Yes	Yes	Yes	Yes
19	Muthanandam et al. 2021 [32]	Yes	Yes	No	No	Yes	Yes	Yes	Yes	Unclear
20	Muthanandam et al. 2022 [33]	No	No	Yes	No	Yes	Unclear	Unclear	Yes	Unclear
21	Rajkuwar et al. 2021 [34]	Yes	Yes	Yes	No	Yes	Yes	Yes	Yes	Unclear
22	Rose et al. 2021 [35]	Yes	Yes	Yes	Yes	Yes	Unclear	Unclear	Yes	Unclear
23	Sadath et al. 2022 [11]	Yes	Yes	Yes	Yes	Yes	Yes	Yes	Yes	Yes
24	Sajeev et al. 2018 [36]	Yes	Yes	Yes	Yes	Yes	Yes	Yes	Yes	Yes
25	Saoji et al. 2018 [37]	No	No	Yes	Yes	Yes	Yes	Yes	Yes	Unclear
26	Seshadri et al. 2020 [38]	Yes	Yes	Yes	Yes	Yes	Yes	Yes	Yes	Yes
27	Shrivastav et al. 2018 [39]	Yes	Yes	Yes	Yes	Yes	Unclear	Unclear	Yes	Yes
28	Tushi et al. 2018 [40]	Yes	No	Yes	Yes	Yes	Unclear	Unclear	Yes	Yes

Overview of included studies

**Figure 2:**
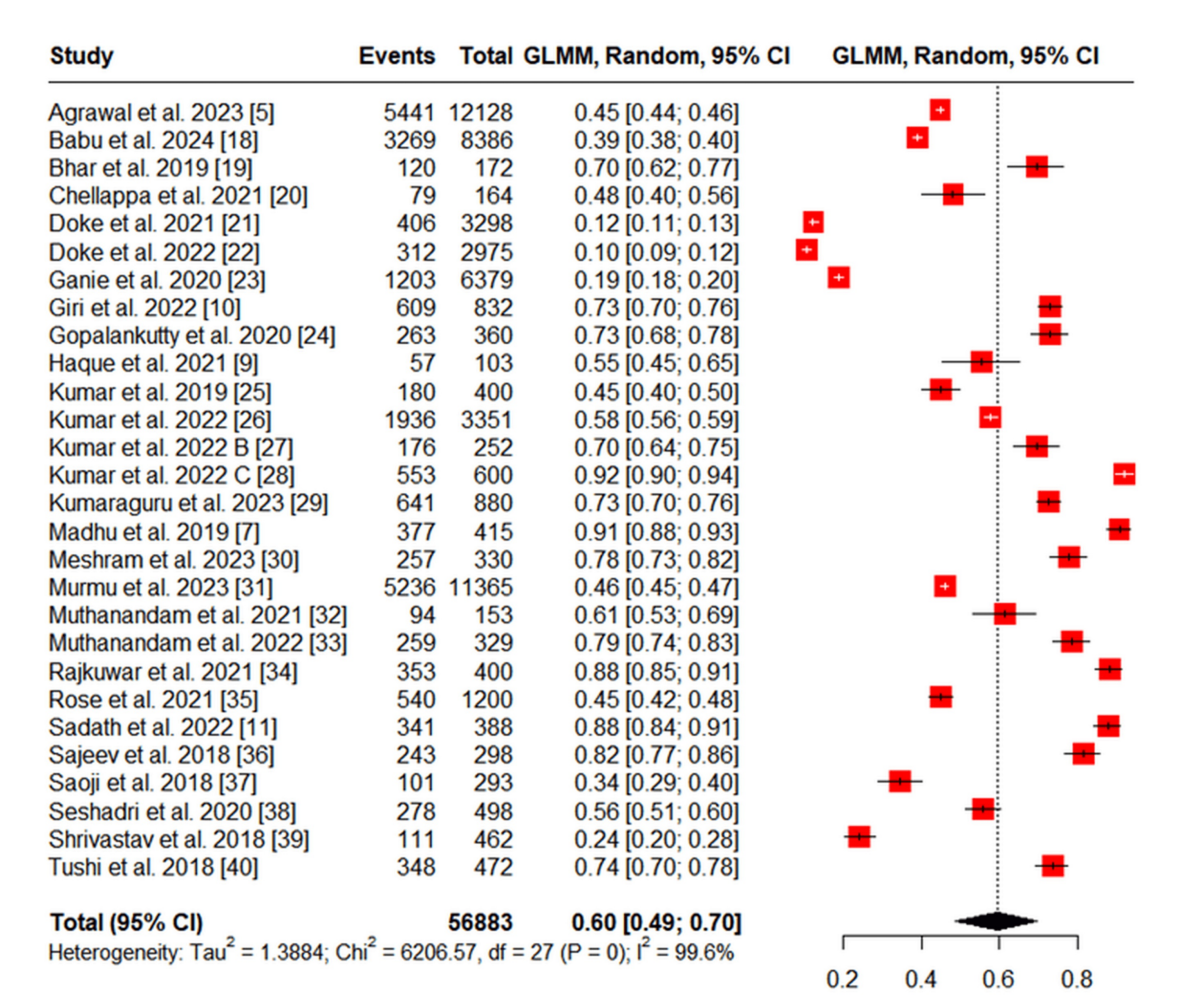
Meta-analysis of Overall Tobacco Use Among Tribal Populations in India GLMM: Generalized linear mixed model

A total of 28 studies were included in this meta-analysis, providing data on the prevalence of tobacco use among various tribal populations across different regions of India (Figure 2). The sample sizes ranged from 103 [9] to 12,128 participants [5]. Of the included studies, 18 reported data on both ST and SLT use, while one reported only on SLT [30], and one focused solely on ST [23]. In seven studies, tobacco use was reported without disaggregation by type. Detailed study characteristics are presented in Table 2.

**Table 2:** Tobacco Use Among Tribal Populations in India: Sample Size, Overall Prevalence, Smokeless and Smoked Forms, and Gender Distribution SLT: smokeless tobacco; ST: smoked tobacco

Sl. No.	Authors and Year	Location (Tribal Group)	Study Design	Study Settings	Sample Size	Total Tobacco Users (%)	SLT users (%)	ST Users (%)	Male Tobacco Users (%)	Female Tobacco Users (%)
1	Agrawal et al. 2023 [5]	Nationwide (Not Specified)	Cross-Sectional	Community Based	12128	5441 (44.86%)	3899 (30.33%)	1542 (12.71%)	2501 (22.00%)	1398 (12.00%)
2	Babu et al. 2024 [18]	Six districts across India (Not Specified)	Cross-Sectional	Community Based	8386	3269 (38.45%)	2187 (25.77%)	1076 (12.68%)	-	-
3	Bhar et al. 2019 [19]	West Bengal (Not Specified)	Cross-Sectional	Community Based	172	120 (70.00%)	107 (62.20%)	44 (25.60%)	-	-
4	Chellappa et al. 2021 [20]	Tamil Nadu (Gypsy)	Cross-Sectional	Community Based	164	79 (48.17%)	50 (63.40%)	23 (29.10%)	37 (53.62%)	42 (71.81%)
5	Doke et al. 2021 [21]	Maharashtra (Not Specified)	Cross-Sectional	Community Based	3298	406 (12.30%)	-	-	-	-
6	Doke et al. 2022 [22]	Maharashtra (Not Specified)	Cross-Sectional	Community Based	2975	312 (10.80%)	-	-	-	-
7	Ganie et al. 2020 [23]	Jammu and Kashmir (Gujjar and Bakarwal)	Cross-Sectional	Community Based	6379	1203 (18.85%)	-	1203 (17.60%)	930 (32.24%)	273 (6.60%)
8	Giri et al. 2022 [10]	Odisha (Sabar and Munda)	Cross-Sectional	Community Based	832	609 (73.20%)	-	-	311 (81.40%)	298 (66.20%)
9	Gopalankutty et al. 2020 [24]	Kerala (Irulas, Mudugas, and Kurumbas)	Cross-Sectional	Community Based	360	263 (73.00%)	-	-	-	-
10	Haque et al. 2021 [9]	West Bengal (Santhal)	Cross-Sectional	Community Based	103	57 (55.50%)	53 (51.00%)	4 (3.80%)	-	-
11	Kumar et al. 2019 [25]	Jharkhand (Birhor)	Cross-Sectional	Community Based	400	180 (45.00%)	134 (33.50%)	46 (11.50%)	-	-
12	Kumar et al. 2022 [26]	Madhya Pradesh (Gond)	Cross-Sectional	Community Based	3351	1936 (57.80%)	1608 (83.10%)	290 (15.00%)	1101 (65.40%)	883 (50.00%)
13	Kumar et al. 2022 [27]	Bihar (Tharus)	Cross-Sectional	Community Based	252	176 (69.80%)	100 (39.60%)	76 (30.20%)	-	-
14	Kumar et al. 2022 [28]	Odisha (Kutia Kandha)	Cross-Sectional	Community Based	600	553 (92.10%)	434 (72.33%)	119 (19.80%)	-	-
15	Kumaraguru et al. 2023 [29]	Tamil Nadu (Irular and Narikuravar)	Cross-Sectional	Community Based	880	641 (73.00%)	353 (40.11%)	288 (32.72%)	-	-
16	Madhu et al. 2019 [7]	Karnataka (Soliga)	Cross-Sectional	Community Based	415	377 (90.80%)	-	-	-	-
17	Meshram et al. 2023 [30]	Chhattisgarh (Not Specified)	Cross-Sectional	Community Based	330	257 (78.00%)	257 (78.00%)	-	-	-
18	Murmu et al. 2023 [31]	Nationwide (Not Specified)	Cross-Sectional	Community Based	11365	5236 (46.10%)	3596 (32.00%)	2115 (19.00%)	-	-
19	Muthanandam et al. 2021 [32]	Puducherry (Narikuravar)	Cross-Sectional	Community Based	153	94 (61.4%)	45 (29.40%)	49 (32.00%)	-	-
20	Muthanandam et al. 2022 [33]	Puducherry (Narikuravar)	Cross-Sectional	Community Based	329	259 (78.85%)	259 (78.85%)	214 (65.23%)	-	-
21	Rajkuwar et al. 2021 [34]	Andaman and Nicobar Islands (Nicobarese Tribes)	Cross-Sectional	Community Based	400	353 (88.25%)	334 (83.50%)	13 (3.30%)	131 (86.18%)	222 (89.51%)
22	Rose et al. 2021 [35]	Tamil Nadu (Malayalis)	Cross-Sectional	Community Based	1200	540 (45.00%)	4 (0.33%)	536 (44.60%)	-	-
23	Sadath et al. 2022 [11]	Kerala (Kattunayakan)	Cross-Sectional	Community Based	388	341 (87.88%)	249 (64.20%)	92 (23.70%)	-	-
24	Sajeev et al. 2018 [36]	Kerala (Kani)	Cross-Sectional	Community Based	298	243 (81.50%)	226 (76.00%)	113 (37.90%)	-	-
25	Saoji et al. 2018 [37]	Northeast India (Tibeto-Burman origin)	Cross-Sectional	Community Based	293	101 (34.40%)	-	-	-	-
26	Seshadri et al. 2020 [38]	Tribal communities living around four tiger reserves (Gonds, Baigas, Nyishis, Aka, Puroik and Soliga)	Cross-Sectional	Community Based	498	278 (56.00%)	-	-	-	-
27	Shrivastav et al. 2018 [39]	Madhya Pradesh (Bharia)	Cross-Sectional	Community Based	462	111 (24.00%)	-	-	75 (30.90%)	36 (16.30%)
28	Tushi et al. 2018 [40]	Nagaland (Ao)	Cross-Sectional	Community Based	472	348 (73.50%)	256 (54.00%)	92 (19.50%)	229 (97.00%)	119 (50.40%)

Pooled prevalence of tobacco use

The overall pooled prevalence of tobacco use among tribal populations in India was estimated at 60% (95% confidence interval (CI): 49%-70%), based on data from 56,883 individuals (Figure 2). A random-effects model was used, and substantial heterogeneity was observed (I² = 99.6%).

Gender-specific prevalence

**Figure 3:**
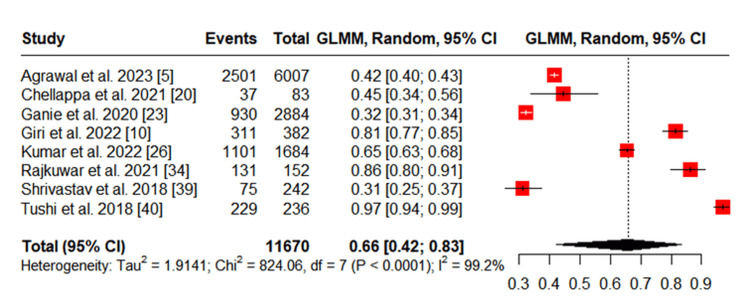
Meta-analysis of Tobacco Use Among Men in Tribal Populations in India GLMM: Generalized linear mixed model

**Figure 4:**
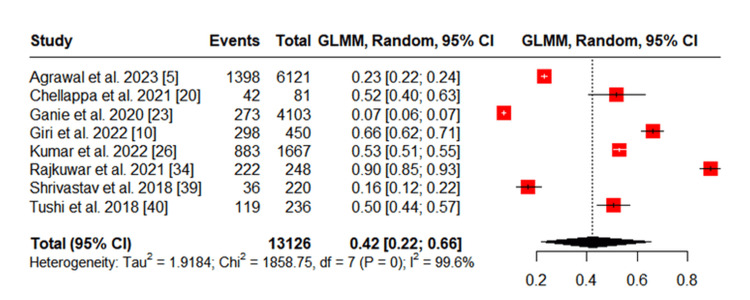
Meta-analysis of Tobacco Use Among Women in Tribal Populations in India GLMM: Generalized linear mixed model

Eight studies provided gender-disaggregated data [5,10,20,23,26,34,39,40]. The prevalence of tobacco use among tribal men ranged from 31% to 97%, while among women, it ranged from 7% to 90%. The pooled prevalence was 66% (95% CI: 42%-83%) among men and 42% (95% CI: 22%-66%) among women, both showing significant heterogeneity (I² = 99.1% for men and I² = 99.6% for women) (Figures 3, 4).

Prevalence by the type of tobacco

**Figure 5:**
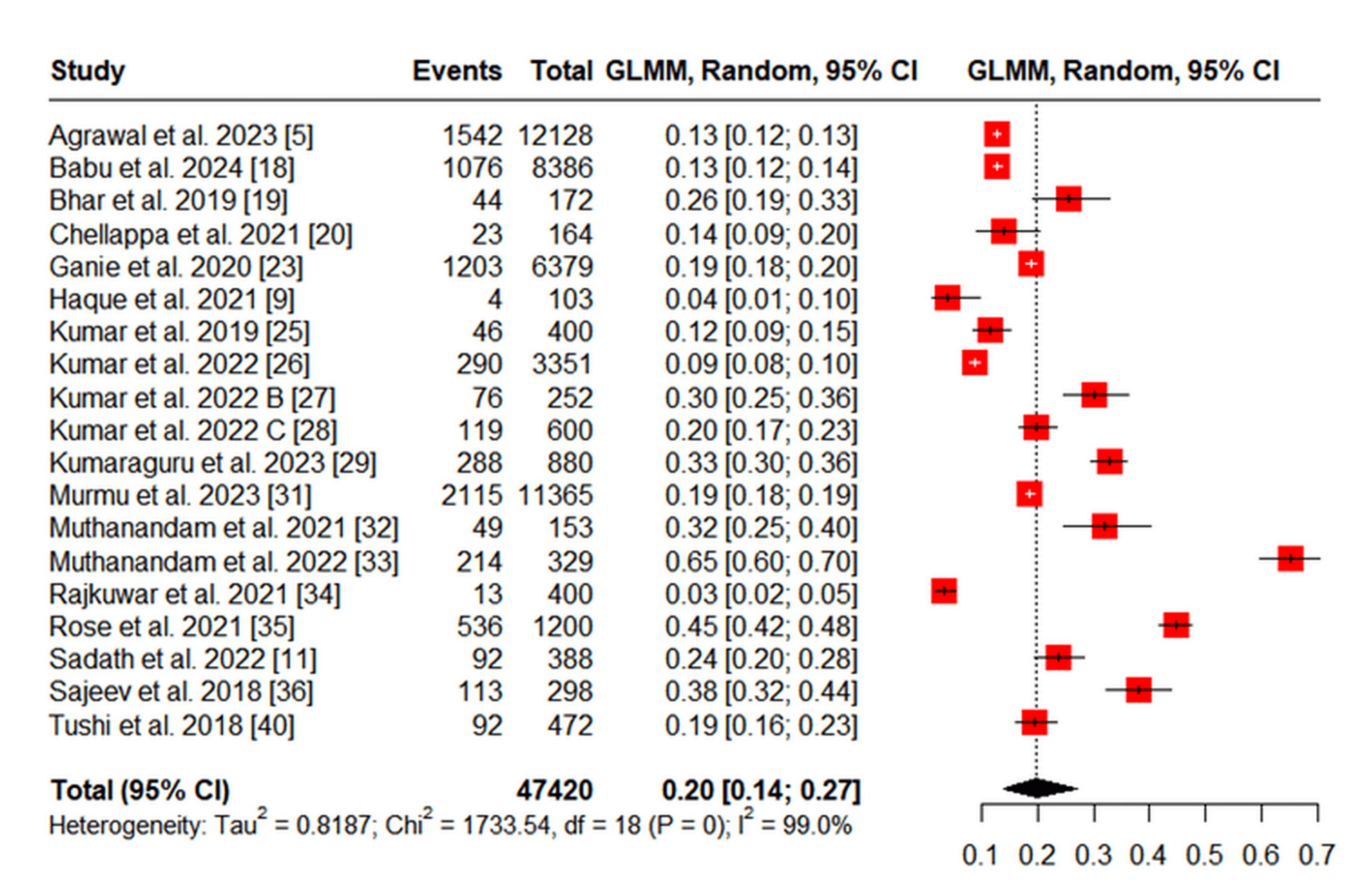
Meta-analysis of Studies Reporting Smoked Tobacco Use Among Tribal Populations GLMM: Generalized linear mixed model

**Figure 6:**
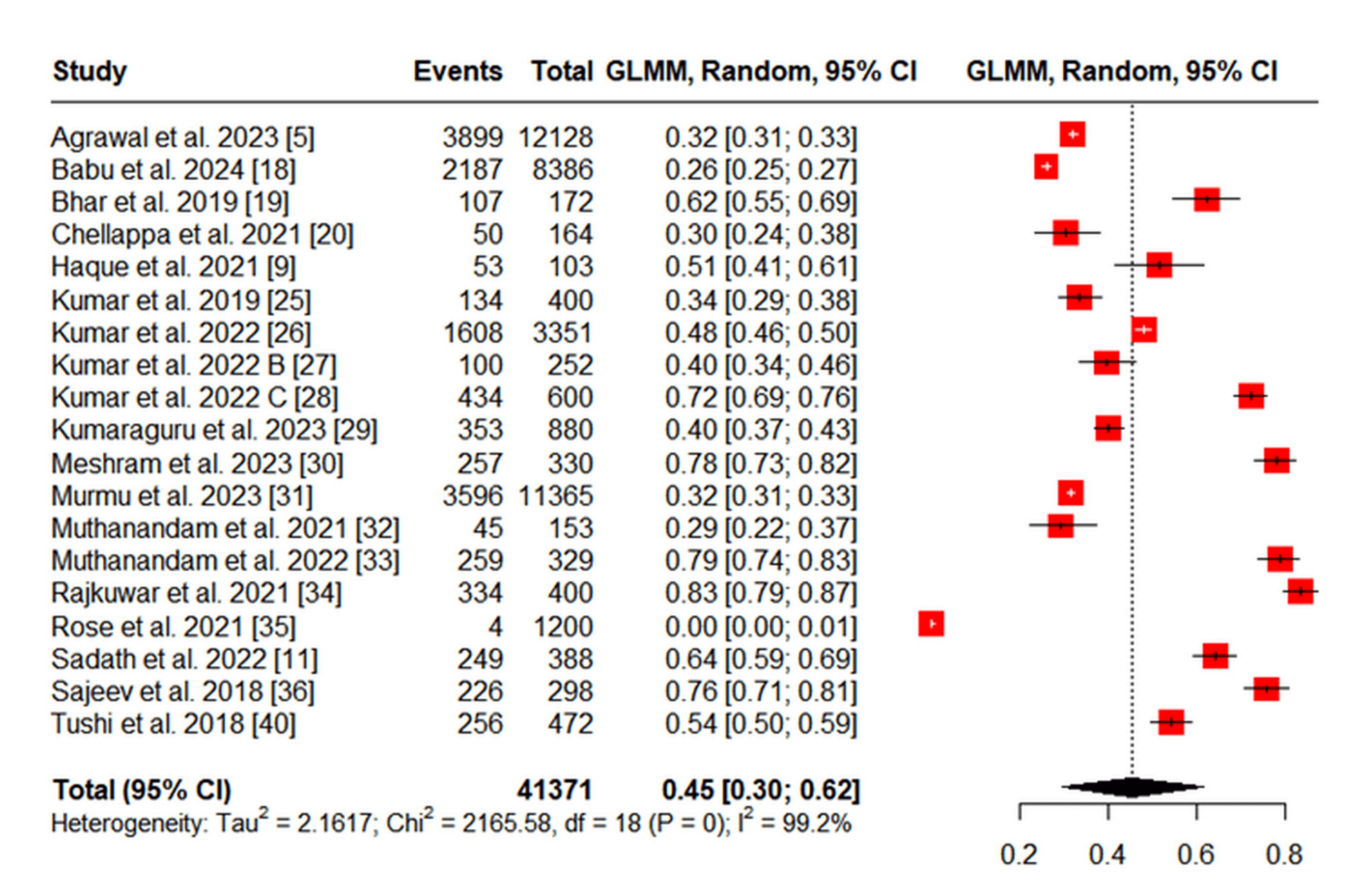
Meta-analysis of Studies Reporting Smokeless Tobacco Use Among Tribal Populations GLMM: Generalized linear mixed model

The pooled prevalence of ST use among tribal populations was 20% (95% CI: 14%-27%), whereas the prevalence of SLT use was 45% (95% CI: 30%-62%), based on a random-effects model (Figures 5, 6). Both estimates also showed high heterogeneity (I² = 99%). These findings indicate that SLT is more commonly used than ST among India’s tribal communities.

Assessment of publication bias

**Figure 7:**
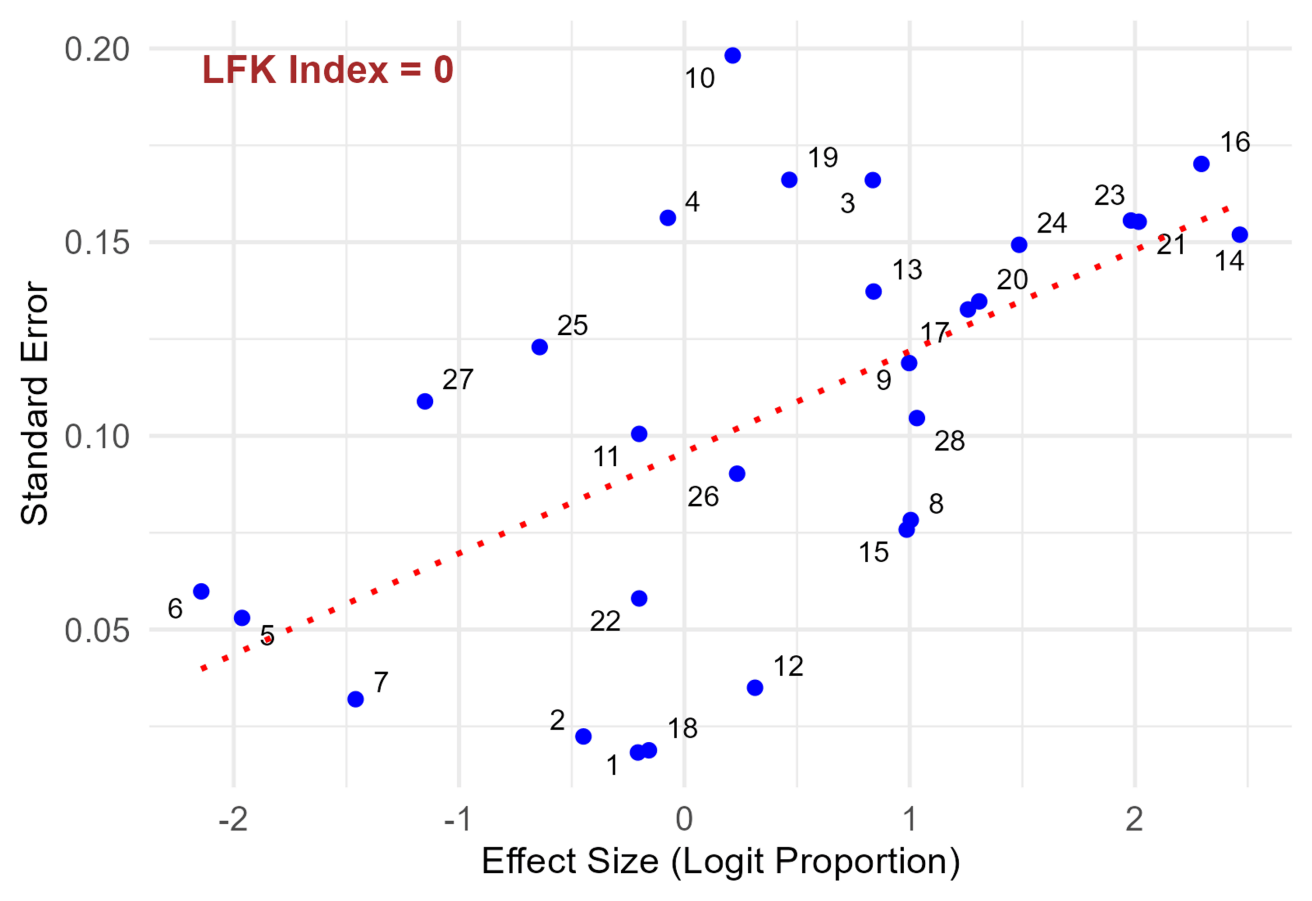
Deviation from the Ordinary Least Squares Regression Line Plot 1: Agrawal et al. 2023 [5]; 2: Babu et al. 2024 [18];  3: Bhar et al. 2019 [19]; 4: Chellappa et al. 2021 [20]; 5: Doke et al. 2021 [21]; 6: Doke et al. 2022 [22]; 7: Ganie et al. 2020 [23]; 8: Giri et al. 2022 [10]; 9: Gopalankutty et al. 2020 [24]; 10: Haque et al. 2021 [9]; 11: Kumar et al. 2019 [25]; 12: Kumar et al. 2022 [26]; 13: Kumar et al. 2022 B [27]; 14: Kumar et al. 2022 C [28]; 15: Kumaraguru et al. 2023 [29]; 16: Madhu et al. 2019 [7]; 17: Meshram et al. 2023 [30]; 18: Murmu et al. 2023 [31]; 19: Muthanandam et al. 2021 [32]; 20: Muthanandam et al. 2022 [33]; 21: Rajkuwar et al. 2021 [34]; 22: Rose et al. 2021 [35]; 23: Sadath et al. 2022 [11]; 24: Sajeev et al. 2018 [36]; 25: Saoji et al. 2018 [37]; 26: Seshadri et al. 2020 [38]; 27: Shrivastav et al. 2018 [39]; 28: Tushi et al. 2018 [40] LFK index: Luis Furuya-Kanamori index

Publication bias was assessed using the deviation from the DOI plot, considered superior to funnel plots for meta-analyses involving proportions [17]. The LFK index was calculated to be 0, indicating no significant asymmetry in the DOI plot and thus suggesting a low likelihood of publication bias among the included studies (Figure 7).

ROB in included studies

**Figure 8:**
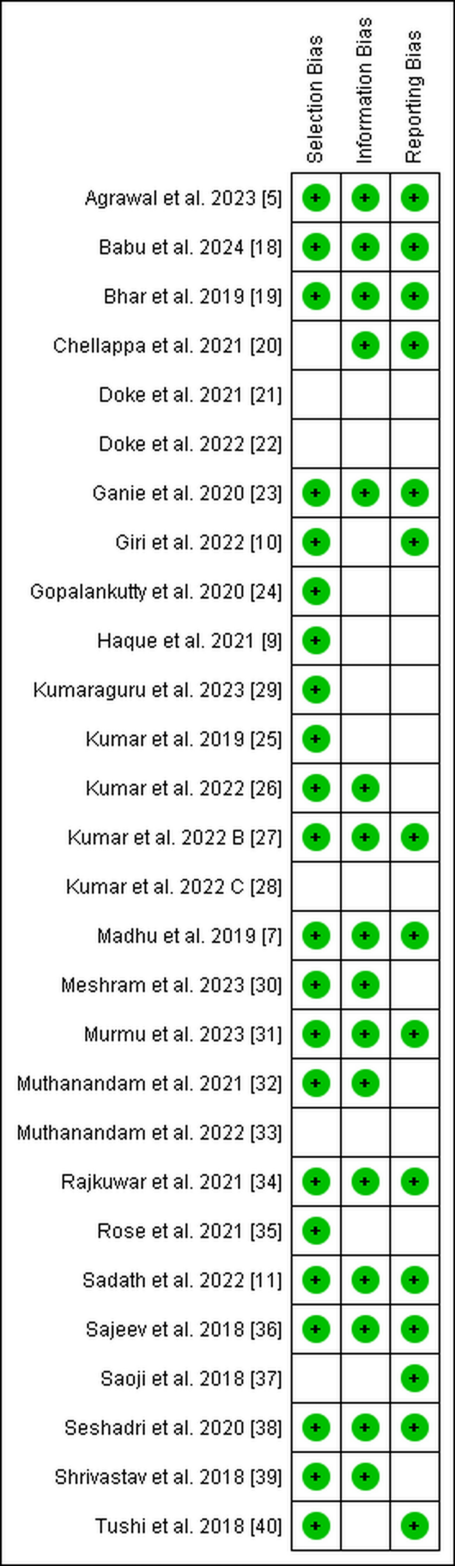
Risk of Bias Evaluation Using RevMan for Included Studies

A majority of studies demonstrated a low risk of selection bias (78.5%), while fewer showed low risk of information bias (57.1%) and reporting bias (53.5%). The remaining studies were categorized as having an unclear risk, primarily due to insufficient methodological reporting (Table 3, Figure 8).

**Table 3:** Assessment of Selection, Information, and Reporting Bias in Included Cross-Sectional Studies on Tobacco Use among Tribal Populations in India

Sl. No.	Authors and Year	Selection Bias	Information Bias	Reporting Bias
1	Agrawal et al. 2023 [5]	Low (Multistage representative random sampling)	Low (Validated GATS 2 Questionnaire)	Low (Operational definition provided)
2	Babu et al. 2024 [18]	Low (Cluster representative random sampling)	Low (WHO Steps Questionnaire)	Low (Operational definition provided)
3	Bhar et al. 2019 [19]	Low (Two-stage representative cluster sampling)	Low (WHO Steps Questionnaire)	Low (Operational definition provided)
4	Chellappa et al. 2021 [20]	Unclear (Not specified any representative sampling)	Low (Fagerstrom Nicotine Dependence Scale)	Low (Operational definition provided)
5	Doke et al. 2021 [21]	Unclear (Not specified any representative sampling)	Unclear (Not specified any questionnaire validation)	Unclear (Not specified any operational definition)
6	Doke et al. 2022 [22]	Unclear (Not specified any representative sampling)	Unclear (Not specified any questionnaire validation)	Unclear (Not specified any operational definition)
7	Ganie et al. 2020 [23]	Low (Multi-stage cluster sampling with probability proportional to size)	Low (WHO Steps Questionnaire)	Low (Operational definition provided)
8	Giri et al. 2022 [10]	Low (Multi-stage cluster sampling with probability proportional to size)	Unclear (Not specified any questionnaire validation)	Low (Operational definition provided)
9	Gopalankutty et al. 2020 [24]	Low (Multistage stratified random sampling)	Unclear (Not specified any questionnaire validation)	Unclear (Not specified any operational definition)
10	Haque et al. 2021 [9]	Low (Simple random sampling without replacement)	Unclear (Not specified any questionnaire validation)	Unclear (Not specified any operational definition)
11	Kumar et al. 2019 [25]	Low (Multi-stage random sampling)	Unclear (Not specified any questionnaire validation)	Unclear (Not specified any operational definition)
12	Kumar et al. 2022 [26]	Low (Simple random sampling)	Low (Pretested tool used)	Unclear (Not specified any operational definition)
13	Kumar et al. 2022 B [27]	Low (Simple random sampling)	Low (Pretested tool used)	Low (Operational definition provided)
14	Kumar et al. 2022 C [28]	Unclear (Not specified any representative sampling)	Unclear (Not specified any questionnaire validation)	Unclear (Not specified any operational definition)
15	Kumaraguru et al. 2023 [29]	Low (Multistage random sampling)	Unclear (Not specified any questionnaire validation)	Unclear (Not specified any operational definition)
16	Madhu et al. 2019 [7]	Low (Simple random sampling)	Low (Pretested tool used)	Low (Operational definition provided)
17	Meshram et al. 2023 [30]	Low (Multi-stage cluster sampling with probability proportional to size)	Low (Pretested tool used)	Unclear (Not specified any operational definition)
18	Murmu et al. 2023 [31]	Low (Multistage representative random sampling)	Low (Validated LASI Questionnaire)	Low (Operational definition provided)
19	Muthanandam et al. 2021 [32]	Low (Systematic random sampling)	Low (Pre-validated questionnaire used)	Unclear (Not specified any operational definition)
20	Muthanandam et al. 2022 [33]	Unclear (Not specified any representative sampling)	Unclear (Not specified any questionnaire validation)	Unclear (Not specified any operational definition)
21	Rajkuwar et al. 2021 [34]	Low (Simple random sampling)	Low (Pre-validated questionnaire used)	Low (Operational definition provided)
22	Rose et al. 2021 [35]	Low (Simple random sampling with probability proportional to size)	Unclear (Not specified any questionnaire validation)	Unclear (Not specified any operational definition)
23	Sadath et al. 2022 [11]	Low (Multistage cluster random sampling)	Low (Pre-validated questionnaire used)	Low (Operational definition provided)
24	Sajeev et al. 2018 [36]	Low (Multistage cluster random sampling)	Low (WHO Steps Questionnaire)	Low (Operational definition provided)
25	Saoji et al. 2018 [37]	Unclear (Not specified any representative sampling)	Unclear (Not specified any questionnaire validation)	Low (Operational definition provided)
26	Seshadri et al. 2020 [38]	Low (Multistage cluster random sampling)	Low (Pre-validated questionnaire used)	Low (Operational definition provided)
27	Shrivastav et al. 2018 [39]	Low (Multistage cluster random sampling)	Low (Pre-validated questionnaire used)	Unclear (Not specified any operational definition)
28	Tushi et al. 2018 [40]	Low (Cluster sampling with probability proportional to size linear systematic sampling	Unclear (Not specified any questionnaire validation)	Low (Operational definition provided)

Main findings

The present meta-analysis reveals that the overall prevalence of tobacco use among tribal populations in India is 58%, which is significantly higher than the national average. Notably, SLT use (45%) was twice as prevalent as ST use (20%). Gender-specific analysis showed that tobacco use was higher in men (66%) than in women (42%). These estimates highlight the disproportionate burden of tobacco use among tribal communities and point to an urgent need for culturally tailored tobacco control strategies.

Comparison With National Data

The National Mental Health Survey (NMHS 2015-16) reported tobacco use prevalence in India at 20.9%, with a treatment gap of 91.8% [3]. The Global Adult Tobacco Survey (GATS-2, 2016-17) estimated overall tobacco use at 28.6%, with 10.7% for smoked and 21.4% for smokeless forms [4]. These national-level estimates are considerably lower than those observed in this meta-analysis. The findings underscore a clear disparity between tribal and general populations, emphasizing the need for population-specific policies.

Comparison With International Data

Several international studies reinforce the global trend of high tobacco use among tribal or indigenous groups. Goeckner et al. (2024) found 29.5% cigarette use, 35.2% e-cigarette use, and 20% poly-tobacco use among American Indian tribal college students [41]. Panaretto et al. (2008) reported 66.6% current tobacco use among Indigenous antenatal mothers in Townsville, Australia-especially in those aged below 20 years [42]. Pedroza-Buitrago et al. (2020) documented a 28% tobacco use prevalence among indigenous adolescents in the Colombian Amazon [43]. Tillery et al. (2024) reported a 53% prevalence of nicotine use among the Cheyenne River Sioux Tribe in the U.S. [44]. These studies, though geographically diverse, echo the elevated tobacco burden among tribal groups, consistent with the findings from the present review.

Gender-Wise Comparison With National Estimates

NFHS-5 (2019-2021) reported tobacco use prevalence of 38% in men and 8.9% in women nationally, and 50.6% in Scheduled Tribe men versus 19.2% in women [45]. GATS-2 reported current tobacco use at 42.4% in men and 14.2% in women [4]. Compared to this, our meta-analysis shows a significantly higher prevalence in tribal men (67%) and women (42%). These differences may stem from methodological variations. NFHS-5 and GATS-2 relied on household-level community-based enumeration, whereas the studies in this meta-analysis included both community- and hospital-based tribal participants. Moreover, the “Scheduled Tribe” classification in national surveys may not capture the diversity and specificity of tribal identities represented in the included studies.

ST and SLT Comparison With Other Studies

Murmu et al. (2023), using data from the Longitudinal Ageing Study in India (LASI, wave 1), found an overall tobacco use prevalence of 46.1% among tribal individuals, with 18.6% smokers and 31.7% SLT users [31]. Other regional studies also show higher SLT prevalence, such as in the Narikurava tribe in Puducherry, where 78.85% reported SLT use and 65.23% ST use [33], and in the tribal population of Bastar, Chhattisgarh, where 78% used SLT [30]. Compared to GATS-2 (ST: 10.7%, SLT: 21.4%) [4], the findings from these regional studies and the current meta-analysis indicate a substantially higher burden of SLT use in tribal populations.

In contrast, international studies show far lower SLT prevalence among tribal groups. Fakunle et al. (2024) reported only 0.54% non-cigarette tobacco use (including SLT) among indigenous African people [46]. Merkin et al. [47] found just 0.2% SLT use among the Kola Sami tribe in Russia, whereas lifetime SLT use was higher in Native American groups from the Northern Plains (31%) and Southwest tribes (30%) [48]. These variations may be attributed to differences in cultural practices, tobacco product availability, and survey methodologies, particularly the framing of SLT-related questions.

Sources of Heterogeneity

High heterogeneity (I² ≈ 99%) was observed across all pooled estimates. This can be attributed to variations in study design, objectives, population characteristics, tribal subgroups, sampling methods, and how prevalence was defined and measured. The socio-demographic diversity among tribal communities and differing contextual factors across Indian states may have also contributed. Although such heterogeneity limits the generalizability of the pooled estimates [49], this review remains valuable as the first meta-analysis to systematically quantify tobacco use prevalence in India’s tribal population. These findings are critical for designing targeted and inclusive tobacco control policies to address this neglected public health challenge.

## Conclusions

This study highlights the high prevalence of tobacco use among tribal populations in India when compared to the general population. The use of SLT is nearly twice as common as ST in these communities. While this pattern aligns with national trends, the extent of use in tribal populations is substantially greater and carries serious health implications. Tobacco use is intricately woven into the socio-cultural practices of tribal communities, where it is often used in festivals, marriages, religious ceremonies, and traditional medicine. This cultural acceptance contributes to early initiation and sustained use, increasing the risk of dependence. Such dependence heightens vulnerability to both communicable and non-communicable diseases, including those affecting oral, respiratory, and cardiovascular health, as well as mental well-being.

Tackling this widespread and culturally rooted behavior needs a thoughtful public health approach. Efforts should be tailored to local contexts, respect cultural beliefs, and involve tribal communities. Key steps include health education, easy access to quitting support, and policies that address harmful traditional practices. By focusing on this high-burden group, India can make meaningful strides in reducing health disparities and improving overall health outcomes. Prioritizing tobacco control among tribal populations also represents a crucial step toward achieving the broader national health objectives and the SDGs by 2030.
